# Which Cognitive Abilities Make the Difference? Predicting Academic Achievements in Advanced STEM Studies

**DOI:** 10.3390/jintelligence6040048

**Published:** 2018-10-26

**Authors:** Michal Berkowitz, Elsbeth Stern

**Affiliations:** Institute for Research on Learning and Instruction, ETH Zurich, CH-8092 Zurich, Switzerland; stern@ifv.gess.ethz.ch

**Keywords:** spatial ability, STEM, intelligence, cognitive abilities, advanced math, higher education

## Abstract

Previous research has shown that psychometrically assessed cognitive abilities are predictive of achievements in science, technology, engineering and mathematics (STEM) even in highly selected samples. Spatial ability, in particular, has been found to be crucial for success in STEM, though its role relative to other abilities has been shown mostly when assessed years before entering higher STEM education. Furthermore, the role of spatial ability for mathematics in higher STEM education has been markedly understudied, although math is central across STEM domains. We investigated whether ability differences among students who entered higher STEM education were predictive of achievements during the first undergraduate year. We assessed 317 undergraduate students in Switzerland (150 from mechanical engineering and 167 from math-physics) on multiple measures of spatial, verbal and numerical abilities. In a structural equation model, we estimated the effects of latent ability factors on students’ achievements on a range of first year courses. Although ability-test scores were mostly at the upper scale range, differential effects on achievements were found: spatial ability accounted for achievements in an engineering design course beyond numerical, verbal and general reasoning abilities, but not for math and physics achievements. Math and physics achievements were best predicted by numerical, verbal and general reasoning abilities. Broadly, the results provide evidence for the predictive power of individual differences in cognitive abilities even within highly competent groups. More specifically, the results suggest that spatial ability’s role in advanced STEM learning, at least in math-intensive subjects, is less critical than numerical and verbal reasoning abilities.

## 1. Introduction

Cognitive predictors of success in the domains of science, technology, engineering and mathematics (STEM) have been a focus of numerous studies. While indicators of general intelligence have been strongly linked with educational attainment in math and science [1], many studies suggest that the cognitive profile of STEM learners goes beyond high general reasoning ability [2,3,4]. Along with mathematical competence, spatial ability is among the cognitive factors that were identified as markers for success in STEM and as a core cognitive resource for STEM learning. Large-scale studies in U.S. populations revealed that high spatial ability (SA) predicted choice and long-term achievements in STEM beyond verbal and quantitative abilities, both among highly selective groups and in more heterogeneous samples [4,5,6]. Indeed, considerable research has examined spatial thinking in various STEM disciplines such as engineering [7,8], chemistry [9], physics [10], geology [11,12] and medicine [13]. Evidence has also accumulated supporting the utility of SA training, which is regarded as a potentially efficient way to increase young students’ chances of entering and remaining in STEM domains [14,15].

Nevertheless, several questions remain open concerning the extent to which SA supports STEM learning across levels and areas of education. For example, reviewing expert-novice studies in several domains, Uttal and Cohen [16] concluded that psychometrically assessed SA is critical for STEM novices (i.e., during the first 2–3 semesters as STEM majors), but becomes less critical with increasing expertise. At the same time, other findings show that individual differences in SA, especially at the upper scores range, predicted outcomes clearly beyond the novice level [17]. As described next, we addressed two issues relevant to the understanding of the SA–STEM link that have not yet received much attention. The first was whether SA predicts STEM achievements beyond other factors when assessed among students already enrolled on STEM programs. The emphasis here was on ability differences within an expectedly high-ability sample of STEM undergraduates, yet not as selected as in other studies (e.g., the top 0.5% and 0.01% in [18]). The second issue was the degree to which SA predicts achievements across STEM areas. The focus here was on advanced mathematics and math-intensive areas such as physics, which are central in STEM education. Thus, we studied the population for whom SA is expected to be particularly important: students who have just begun their undergraduate studies in STEM. Our study was therefore a-priori targeted at students who select advanced STEM programs rather than at generalizing to math and STEM learning more broadly. We next describe each of the above issues in more detail.

### 1.1. The Unique Role of Spatial Ability in STEM and What Spatial Ability Tests Measure

Very broadly, spatial ability (SA) refers to “the ability to generate, retain, and manipulate abstract visual images” [19]. In fact, several types of SA have been identified since the early factor analytic studies on human cognitive abilities [20,21], and the exact distinctions vary between studies and theoretical frameworks [22,23,24,25,26]. The most studied type of SA in the context of STEM is spatial visualization (SV), which is the ability to “…apprehend a spatial form, shape or scene in order to match it with another spatial form, shape or scene, often with the necessity of rotating it in two or three dimensions one or more times” [24]. Typical tests reflecting this factor are *The Paper Folding Test* [27] and *The Mental Rotations Test* [28,29]. Others have differentiated mental rotation from SV [25], defining the former as involving an analogue and holistic mental process and the latter as involving more complex, multistep mental manipulations. Measures of both kinds have been extensively used in previous research on STEM learning [30], and therefore were in focus of the present research as well. We use the term SV in the broader sense suggested by Carroll [24], namely referring to complex mental manipulations of objects, mostly in three dimensions, involving either rotation or other forms of transformation. Since previous studies have often used the broad term SA even when measures were of SV, we use the term SA when referring to previous research in this field.

Cognitive ability tests are multidimensional to varying degrees, and SA tests are no exception. Research showed that solving SA tests involves not only strictly spatial-visual processes, but also a variety of non-spatial components. For example, Miyake et al. [31] showed that performance on SA tests, and particularly on SV tests, highly depended on executive functions, whereas others found that analytical- and heuristic-based strategies are as common and at least as efficient as visualization per se, sometimes even preferable [9,30,32,33]. These components may be considered domain-general in nature, and potentially present in other types of ability tests. Consequently, both visual-spatial and domain-general factors may drive a link between SA and STEM achievements, a distinction that is important to the way this link is understood. The strongest evidence for the unique predictive validity of SA for STEM achievements (i.e., not explained by other abilities) is based on ability differences assessed before entry into higher-education STEM tracks. For example, both math and SA assessed before age 13 predicted very long-term STEM outcomes such as advanced degrees and tenure positions, and these predictions held even among extremely high-ability adolescents [18,34]. It is reasonable to assume that those who have reached the top in STEM careers have done very well also earlier on, for example, during their undergraduate years. If certain abilities were critical for reaching those distal outcomes, then it is also reasonable to expect that they would play a role in achievement prediction earlier in the academic track. However, the interplay between cognitive abilities among students who select STEM at the undergraduate level has been less systematically studied for its impact on achievements. For example, many of the studies that focused on the way SA is related to performance in specific domains in higher STEM education have not accounted for other relevant cognitive abilities that may influence this relation (e.g., Sorby [35] in engineering; Stieff et al. [9] in chemistry). Among studies that included non-spatial measures, for example with dentistry students [13], SA predicted performance on very specific and spatially rich subjects (restorative dentistry), but not more broadly (anatomy grades or the grade point average). Other studies focused on advanced STEM learning among non-STEM participants [10,36], who may not represent the population of STEM beginners. Finally, at least in the area of engineering, the focus has been stronger on students with initially low SA [7,8] than on individual differences at higher levels of SA. Thus, at the time point of beginning undergraduate STEM studies, it is less established whether having exceptional spatial skills continues to affect achievements beyond other abilities.

### 1.2. The Role of Math in STEM Programs

Although the acronym ‘STEM’ has become a notion in itself, it in fact stands for a wide range of knowledge domains. In some cases, a straightforward similarity exists between problems in SA tests and domain-knowledge problems (e.g., switching between two- and three-dimensional representations of objects), and studies in areas such as engineering and geology [8,11] support this observation. However, such similarity is not obvious in all STEM areas. Our primary interest was in mathematics for STEM beginners. Not only is math a STEM discipline itself, it is also central to study programs in all STEM domains, and it is integral to studying other areas such as physics. Moreover, math is one of the most abstract and demanding areas in higher STEM education, posing great difficulties to many students and often crucial to their overall success. In spite of its importance, little is known about the role of SA for math at this level. Our own review [37] revealed that whereas a ‘math-space’ link [38] seems established for fundamental forms of mathematical thinking (e.g., early number representation) [39], evidence for a unique spatial-math link becomes mixed as early as the beginning of formal education [3,40,41,42,43] and all the more so with respect to undergraduate math [36,44,45,46]. Furthermore, math in higher STEM education is, to a great extent, expressed in symbolic language. Although ideas may be represented externally in visuo-spatial form (e.g., shapes of functions), problem solving primarily relies on calculation, proof, and rigorous mathematical argumentation (i.e., not relying on the senses). These might be at odds with problems in SA tests, which typically simulate familiar physical experiences (e.g., rotating objects) and are often designed to activate an analogue mental code. In fact, other types of processes in solving SA tests, which we have termed ‘domain general’ (i.e., analytical, heuristic based etc.), may have more in common with abstract math thinking than visualization itself. Thus, it was unclear to what extent high spatial skills contribute to math learning at this advanced level.

### 1.3. The Present Study

We were concerned with the interplay between cognitive abilities and success in specific topics during higher STEM education, and particularly with the role of SA. We assumed that any specific ability that serves as a critical cognitive resource for advanced STEM learning should have unique predictive validity for achievements also when assessed among students enrolled on advanced STEM studies. Consistent with previous research, we focused our investigation on SV ability. Specifically, we examined whether SV predicted achievements in mathematics, math-intensive subjects, and in typical ‘spatial’ areas in the course of higher STEM education, and whether it was predictive beyond verbal and numerical reasoning abilities. We focused on STEM beginners: students at their first undergraduate year in a STEM program, for whom SA is expected to be critically important. Since this population was likely to be a high-ability group due to factors such as self-selection and admission criteria, our investigation also challenged the notion that ability differences cease to matter in higher levels of performance (the so-called “threshold hypothesis” [47]). Building on the findings cited above, as well as on other findings against a ‘threshold’ view of cognitive abilities [48,49], it was reasonable to expect that variability in cognitive abilities among STEM students will be relevant to achievement prediction. To this end, we examined the predictive validity not only of SV, but also of verbal and numerical reasoning abilities. Our outcome variables were achievements on the most important courses during the first undergraduate year, those that largely determine retention. Focusing on course performance (mostly in the form of grades) as a criterion variable has been done previously in studies on STEM learning [7,35,44]. The main advantage of this approach is the proximity of the outcome variables both in time and in content to the type of learning in question, while keeping external validity high. Nonetheless, some of the studies cited above focused on other types of outcomes, often more distal ones (e.g., [34]), a point to which we return in the discussion.

We investigated the following questions:

1. Does SV ability predict achievements on major STEM courses during the first undergraduate year beyond numerical and verbal reasoning abilities?
2. Does SV ability differ in its predictive validity between domains of achievements (i.e., mathematics or math-intensive areas vs. explicitly spatial domains)?

We systematically investigated these questions by differentiating between ability domains, achievement domains, and STEM programs. Additionally, we included multiple ability measures in order to ensure that the results are not dependent on a particular type of ability test. We assumed that different factors in SV test performance could underlie its link with achievements, namely factors specific to the spatial modality or domain-general ones. The higher the spatial-demands in a field, the stronger we expected its link with spatial-specific factors to be. 

## 2. Method

In a prospective cohort design, we assessed students from two STEM programs on multiple measures of SV, numerical and verbal reasoning abilities. Students’ grades on a range of first year courses served as measures of academic achievements and were based on scores in written exams. Cognitive abilities were modelled as latent variables (LVs), which enabled the direct estimation and control of measurement error across the different tests (e.g., [50]). The effects of abilities on academic achievements were then estimated in a structural equation model (SEM). Since our focus was on the interplay between specific abilities in achievement prediction (i.e., their unique predictive validity), we first specified a correlated factors model with three latent variables: verbal reasoning, numerical reasoning and spatial visualization (SV). We then estimated the effects of these variables on academic achievements, which indicated the extent to which each ability contributed to the prediction over and above the other abilities in the model. Next, in order to directly estimate the contribution of domain-general factors, we examined whether and to what extent specific abilities predicted achievements beyond a general ability factor. 

Latent variables analyses were conducted in M*Plus* (version 7.11; [51]). In all analyses, robust maximum likelihood estimation (MLR) was used, which takes into account non-normal distributions. We followed recommendations described by Kline (2016) for estimating model fit: Exact fit: (1) Model Chi-square with its degrees of freedom and *p* value. Approximate fit indices: (2) Steiger-Lind Root Mean Square Error of Approximation (RMSEA); [52], and its 90% confidence interval (3) Bentler Comparative Fit Index (CFI); [53] (4) Standardized Root Mean Square Residual (SRMR).

### 2.1. Sample

Participants were students in their first undergraduate year at ETH Zurich, which is a large public technological university in Switzerland, and of high reputation internationally. As in many European universities, admission to ETH requires no admission exams and only a negligible tuition fee, though a Gymnasium diploma is required. In the Swiss school system, the Gymnasium is the highest track of secondary school, designed to prepare students for university education. Selection to the Gymnasium starts in middle school (6th grade) or lower secondary school (8th or 9th grade). About 20–25% of an age group eventually attend this academic school track. To this end, we had grounds to expect our sample to be above average in their cognitive abilities. At the same time, as a public university that serves all Gymnasium graduates, ETH attracts a large number of students (e.g., graduation rates across the engineering bachelor programs are over 500 students per year). Students differ in their educational background because although Swiss Gymnasium schools teach the same core subjects, variation exists in their foci and the diploma is based on exams that are not nationally standardized. Additionally, roughly 20% of students beginning bachelor studies at ETH come from abroad (most of them from other German speaking countries, where admission to the Gymnasium is typically less selective than in Switzerland). Finally, the first undergraduate year at ETH is a critical one for retention: students undergo challenging exams with a failure rate of 30%. Thus, while we expected a higher-than-average-ability sample, we did not expect an extremely selected or gifted group. Considering that a large body of research in this area has been conducted in an American population, it is noteworthy that differences between educational systems may complicate the comparability of samples. For example, unlike in Switzerland, in the U.S. there is no equivalent selection process to secondary school, but there are admission exams that lead to large differences between universities in students’ cognitive preconditions. This is less the case in Europe, particularly in smaller countries with only few universities. Additionally, university and college students in the U.S. usually select their major area at a later time point during higher education than students in European universities. Higher education in Switzerland resembles that of other European countries in many respects, and thus sampling from ETH should allow generalizations beyond the institute itself, presumably to high-level technological institutes elsewhere.

We recruited students from bachelor programs in mechanical engineering, mathematics and physics. Math and physics students have an identical curriculum during the first undergraduate year and therefore formed one group in this study, while engineering students formed the second group. There were several reasons for selecting these groups. First, in both groups, math courses are highly intensive during the first year and have a major weight in the overall grade. The major math courses in both groups are analysis (calculus) and linear algebra, thereby providing consistency with respect to the broad mathematical areas in question. At the same time, these math courses are taught differently in each group (e.g., applied versus theoretical), since mathematics is required for different purposes in these study programs. Second, we were interested in comparing math achievements with achievements in more typical ‘spatial’ areas, which we expected to be present at least in the study program of mechanical engineers. Third, both study programs include intensive physics courses that are both mathematically demanding and potentially involve spatial demands. Finally, we were interested in STEM areas that are central and exist as study programs in similar institutes worldwide. 

Participation criteria were as follows: a first-year first-semester student in either math, physics or mechanical engineering; a native (or native level) German speaker; not repeating any of the first year courses (i.e., due to previous failure); and having taken a self-evaluation test in mathematics, which is offered at the beginning of each academic year to all new students. This math test assesses high-school level math knowledge and was included in order to address additional research questions not discussed in this paper. 

*Sample size*: We planned to conduct our main analyses separately in each of the study groups, since courses differ between study programs. Prior simulation studies indicated that in order to detect effects of about 0.3 (latent level) with a correlated factors model (i.e., path coefficients from any ability factor to an outcome), a minimum of about 150 observations would be needed in each group. Therefore, we aimed to reach at least this sample size. 

### 2.2. Measures

The study included multiple measures of cognitive abilities, which were to serve as indicators for latent variables of these abilities. In addition to SV tests, we administered an intelligence test battery with verbal, numerical and figural subscales. This enabled us both to investigate the incremental predictive validity of SV, as well as to estimate the ability range of our sample relative to existing norms. The study also included multiple measures of working memory, which are related to additional research questions not discussed here. 

#### 2.2.1. Spatial Visualization Tests

*The Paper Folding Test* [27]. In this test, participants saw a drawing of a folded piece of paper in which holes were made. They were asked to imagine what the paper would look like when unfolded, and to decide which of the five shapes corresponds to the resulting image. The test consisted of 20 questions divided into two parts of 10 questions each. In line with the standard procedure described in Ekstrom et al. [27], participants were given 3 min to complete each part. Scores on the test were the total of correct answers and ranged from zero to 20. 

*Mental Rotations Test.* This test was the Peters et al. [54] version of the original paper and pencil Mental Rotations Test by Vandenberg & Kuse [28]. In this test, participants were shown a drawing of a cubical figure and had to decide which two other figures out of four were rotated versions of the target figure. In all cases, the target figure appeared on the left and the answer choices on the right. Only two answers were identical to the target, but rotated along the y-axis. The two other answers were mirror images of the target and thus could not become identical to the target by rotation. There were 24 questions divided into two parts of 12 questions each. In line with the procedure described by Peters et al., participants were given 3 min to complete each part. An answer was scored as correct only if both rotated figures were identified. Scores were the total of correct answers and ranged from zero to 24. 

*Mental Cutting Test (MCT).* This is a sub-set of CEEB Special Aptitude Test in Spatial Relations, developed by the College Entrance Examination Board, USA [55]. In this test, participants were shown a drawing of a 3D shape being cut by a plain. Their task was to decide which of five alternatives was the resulting 2D cross-section. There were 25 questions in this test. In line with the standard procedure, participants were given 20 min to complete the test. Scores were the total of correct answers and ranged from zero to 25. 

*The mental cutting test “Schnitte”.* This test was developed by Fay and Quaiser-Pohl [56] (see also [57]) and focuses on the visualization of cross-sections. It was designed especially for identifying exceptionally high spatial ability, and therefore it is a highly difficult test. As we expected our students to be of high ability, we were particularly interested in including this test. The test consists of 17 multiple-choice questions in which participants are asked to visualize various types of cross-sections. Some questions display a 3D shape that is to be cut by a plane or by a 3D shape, and participants need to decide which answer out of five alternatives corresponds to the resulting cross-section. Another type of question shows a resulting cross-section, and participants are required to decide from which combination of shapes out of five alternatives the cross-section could result. Finally, some of the questions were presented only verbally (i.e., no drawings were provided) so that participants had to mentally construct the entire problem. Participants were given 30 min to complete the test. Scores on the test were the total of correct answers and ranged from zero to 17. 

The following two tests are taken from the intelligence test battery IST 2000R (described in more detail below):

*Figure selection.* In this test, participants saw five images of two-dimensional shapes and ten arrays of these shapes ‘torn’ into pieces. Participants were asked to decide which of the whole objects results from putting together the pieces in each array. The test is essentially a two-dimensional object assembly task, which we assumed strongly involve SV. Therefore, we included this task as an indicator for an SV factor. 

*Cube task.* In this test, five drawings of a cube were shown. Each cube had three visible sides, on which different patterns of dots appeared. Below these, 10 additional cubes were presented, each identical to one of the target cubes but presented in a different orientation. For each of the ten cubes, participants had to decide which of the upper five target cubes is the correct one. This test is expected to involve a high degree of mental rotation, therefore we included it as an indicator of an SV factor. 

#### 2.2.2. Numerical and Verbal Reasoning Tests

##### Intelligence Structure Test

The German intelligence structure test 2000 revised (Intelligenz-Struktur-Test) (I-S-T) 2000R [58] was developed along the theoretical lines of well-established models of intelligence [20,59,60] and has been standardized in German speaking populations. The test measures reasoning ability in three content domains: verbal, numerical and figural. There are three sub-scales per content domain, each consisting of 20 multiple-choice questions at increasing difficulty levels. Sub-scales of verbal reasoning include *sentence completion* (e.g., “the opposite of hope is__?__); *analogies* (forest:tree is like meadow: ?); and *similarities* (which two words out of six have the most in common). Sub-scales of numerical reasoning include arithmetic *calculations* (solving simple equations); *number series* (inferring a rule underlying a series of numbers in order to decide which number is next, e.g., 9 7 10 8 11 9 12 ?); and *numerical signs* (deciding which numerical operations are missing in order for an equation to be correct, e.g.,: 6 ? 2 ? 3 = 5). Sub-scales of figural reasoning include *figure selection* and *cube* that are described above, as well as a figural *matrices* test, which measures inductive reasoning with non-verbal stimuli. This last test was not expected to reflect SV and was therefore not included in the ability models and SEM analysis described next. We included this subscale in order to obtain complete scores on the intelligence battery. Each sub-scale has a time limit of 8 to 10 min. The overall duration of the entire battery is approximately 90 min. Raw scores on each sub-scale were the total of correct answers and ranged from zero to 20. Raw scores per content domain were means of scores on the three sub-scales and ranged from zero to 60. We used raw scores in all of the analyses.

#### 2.2.3. Achievements on STEM Courses

Grading in most of the courses of the first undergraduate year is based on scores in written exams usually taken at the end of the year. The exams are composed by senior faculty members based on standard curricula and are given collectively to the entire cohort. Thus, a final grade in a course is not determined by sources such as exercises or interim evaluations, and therefore reflects success in a final exam. Exam scores are transformed into grades ranging from 1 (worst) to 6 (best) with steps of 0.25 points (i.e., equivalent to a 1–20 scale). We collected grades on some of the major first year courses in each study program. In the mechanical engineering group, these were as follows: *Analysis (calculus), linear algebra, physics (mechanics), machine elements, technical drawing and CAD* (computer-aided design); and in the math-physics group: *Analysis, linear algebra*, two physics courses (*physics I: mechanics, physics II: waves, electricity and magnetism*). Of these courses, *technical drawing and CAD* (*T.D.CAD*) is the only course we could confidently describe as having SV at its focus. The course *machine elements* combines spatial tasks (e.g., learned in *T.D.CAD*) with mechanics knowledge and to some degree mathematics. For the rest of the courses, the degree of spatial material or demand was less clear. Courses considered ‘math-intensive’ were all math and physics courses. The course *machine elements* was the only exception for which grades were based both on an exam as described above, and on a semester project. 

### 2.3. Procedure

Students were recruited across three consecutive cohorts (years 2012, 2013, 2014). The first recruitment was in the framework of a pilot study. In this smaller sample, participants performed all of the paper-and-pencil tests and a digital version of the test *Schnitte*. In the following two years, two larger samples were recruited, which were administered the same paper and pencil tests as well as a paper version of *Schnitte*. Students were recruited at the very beginning of the fall semester and were offered monetary compensation for participation, which was compatible with a standard student’s job (20 CHF per hour, a total of 100 CHF for five hours).

Students were invited to two group-testing sessions. The first session included all of the paper and pencil tests except *Schnitte*, and the second session included working memory tasks (not discussed here) and the test *Schnitte*. The sequencing of tests was fixed for all participants, sessions and samples. For each cohort, we collected students’ grades in final exams during the fall of the following academic year. Final exams on all courses take place in summer, i.e., after two semesters of study, except the exam for *T.D.CAD*, which takes place in winter (after one semester). All participants gave their informed consent for inclusion before they participated in the study.

## 3. Results

### 3.1. Sample

A total of 319 students participated in the study. Two participants were excluded from analysis due to prior experience with first year courses or extreme outlier values on most measures. The final sample size was therefore 317, with 150 mechanical engineering students and 167 math-physics students. Table 1 summarizes sample size across cohorts, study groups and gender. The total number of new students that enrolled on these study programs in the years 2012, 2013 and 2014 was 442, 465 and 426 respectively in mechanical engineering; and 299, 326 and 315 in math-physics. Thus, for each cohort, our sample comprised 8–15% of the students in mechanical engineering and 9% to 26% of the students in math-physics. The proportion of women in our sample matched those usually found in these study programs at ETH. Mean age was 19.5 (SD = 1.4) and ranged from 17 to 25. There were no significant differences between cohorts on age (F_2,314_ = 0.19, *p* = 0.83), proportions in study programs (χ^2^ = 4.29, *p* = 0.12) or proportion of women (χ^2^ = 2.11, *p* = 0.35). There were no significant differences between the three cohorts on any of the study measures, except for a small advantage for students in the pilot sample on the number series test (F_2,311_ = 4.39, *p* = 0.01). Thus, the three cohorts were highly similar on the predictor variables, indicating consistency in self-selection to the study (and to ETH). We therefore aggregated cohorts for further analyses.

### 3.2. Data Screening and Missing Data

Values over 3.5 SDs from the mean were considered outliers and treated as missing values. There were 18 outlier values (0.4% of the dataset). Additionally, 14 scores on the test ‘Schnitte’ were not recorded due to a technical error and were also treated as missing values. Finally, 44 participants (14% of the sample) did not attend final exams and therefore had missing data on grades. Not attending exams could be due to any of the following reasons: drop out (4.7%); switching to another department (1.9%); postponing examination to a later time point (3.5%), or an unknown reason (3.8%). Independent samples *t*-tests (corrected for multiple comparisons) revealed a significant difference in favor of students with grades only on one measure (‘numerical signs’; *t* = 3.76, *p* < 0.001; *d* = 0.60). Altogether, the proportions of missing data on grades was 0.6% in the mechanical engineering group and 1% in the math-physics group (for comparison, analyses in which participants with missing data on grades was excluded are available in the supplementary materials part IV). Full information maximum likelihood (FIML) was used for treating all missing data (the default in Mplus).

### 3.3. Descriptive Statistics

Table 2 presents descriptive statistics of all cognitive measures included in the study. As shown, students’ highest scores were on numerical scales. For most scales, reliability estimates based on Cronbach’s alpha were within an acceptable range, though lower than in more heterogeneous samples. This may be expected since the lower the variability in test scores is, the lower the reliability estimates tend to be [60,61,62]. For the verbal scales, these estimates were considerably lower. However, since the verbal scales yielded lower reliability estimates than numerical and figural IST scales also in the normative sample, we assume that the estimates in our sample resulted from both lower score variability, as well as test properties independent of this sample (e.g., verbal test items being less unitary than numerical and figural items). The verbal scales have, nonetheless, been used extensively before as part of the IST test battery. Also, as described next, we modelled all abilities as latent veriables, which takes into account measurement error in the observed scores. The verbal scales loaded clearly on one factor, and they also showed consistent correlations with some of the outcome measures. Lower reliability estimates have usually been described as resulting in an underestimation of a relationship between the scale and other variables (e.g., [60]). To our view, finding correlations in spite of lower reliability, together with the other points mentioned above, justified retaining these scales in the analysis. We additionally report the general lower bound (GLB) estimate of scale reliability, which has been found to be a more accurate measure of reliability than the alpha coefficient in cases of skewed distributions [63,64].

To determine the ability level of our sample, we compared scores on the intelligence scales to existing norms in a population of high-school graduates and in the general population. Performance in the present sample was above average in both these populations, as shown in Table 3. In general IQ scores, our sample had a mean IQ of 119.71 (*SD* = 11.15) relative to the norms for high-school graduates, and 128.15 (*SD* = 10.72) relative to the general population norms. Thus, the data confirmed that students in our sample were of high general cognitive ability. We assume that this is not solely a result of an early selection process in middle school (as described in the methods section), but also of self-selection to this technological university. 

Norms were additionally available for the test *Schnitte*. Mean performance among general high-school graduates in Germany was 7.9 (*SD* = 3.27), and 8.0 (*SD* = 3.84) among scientific high-school graduates. Thus, performance in the present sample (M = 8.55, *SD* = 3.04) was comparable to the level among students with a scientific high-school background. For the other SV tests (paper folding, mental rotations and mental cutting), norms were not readily available. In comparison to the data reported in previous studies, our sample seems to have performed in the upper range found for students in scientific fields. For example, Peters et al. [54] reported a mean of 14.8 (*SD* = 4.8) on the mental rotations test for 135 males in scientific bachelor programs. Tsutsumi, Schroecker, Stachel, and Weiss [65] reported means on the mental cutting test in the range of 15–18 for students in technological universities.

### 3.4. Group Differences

We examined whether engineering and math-physics students differed on the independent (observed) variables, as such differences might influence the results of grade prediction. Independent samples *t*-tests (corrected for multiple comparisons) revealed small effect-size differences in favor of math-physics students on two measures: Schnitte (M = 8.01, *SD* = 2.78 in engineering; M = 9.06, *SD* = 3.18 in math-physics; *t* = 3.01, *p* = 0.003, *d* = 0.34); and verbal similarities (M = 12.07, *SD* = 2.61 in engineering; M = 13.11, *SD* = 2.41 in math-physics; *t* = 3.67, *p* < 0.001, *d* = 0.41). Differences that did not reach significance were all of small to negligible effect size. Thus, students from these two study programs were more similar than different on the mean level of cognitive ability. 

Finally, although not the focus of this study, some gender differences emerged on the cognitive variables. Independent samples *t*-tests revealed significant differences in favor of men on the *Mental Rotations Test* (Men: M = 16.42, *SD* = 3.88; Women: M = 14.14, *SD* = 3.94; *t* = 3.62, *p* < 0.001, *d* = 0.59); and on *numerical signs* (Men: M = 17.49, *SD* = 2.45; Women: M = 15.91, *SD* = 2.59; *t* = 3.95, *p* < 0.001, *d* = 0.64). Men also tended to outperform women on the *Mental Cutting Test* (Men: M = 19.40, *SD* = 3.86; Women: M = 18.07, *SD* = 3.86; *t* = 2.10, *p* = 0.04, *d* = 0.34); and women tended to outperform men on the *Paper Folding Test* (Men: M = 16.59, *SD* = 2.42; Women: M = 17.66, *SD* = 2.53; *t* = 2.72, *p* < 0.01, *d* = 0.44) and on *figural matrices* (Men: M = 11.47, *SD* = 2.75; Women: M = 12.59, *SD* = 2.43; *t* = 2.56, *p* = 0.02, *d* = 0.42), but these differences did not reach significance levels once correcting for multiple comparisons. There was no indication for gender differences on the other ability tests, academic achievements or drop-out rates. 

### 3.5. Latent Structure of Cognitive Abilities

**Figure 1:**
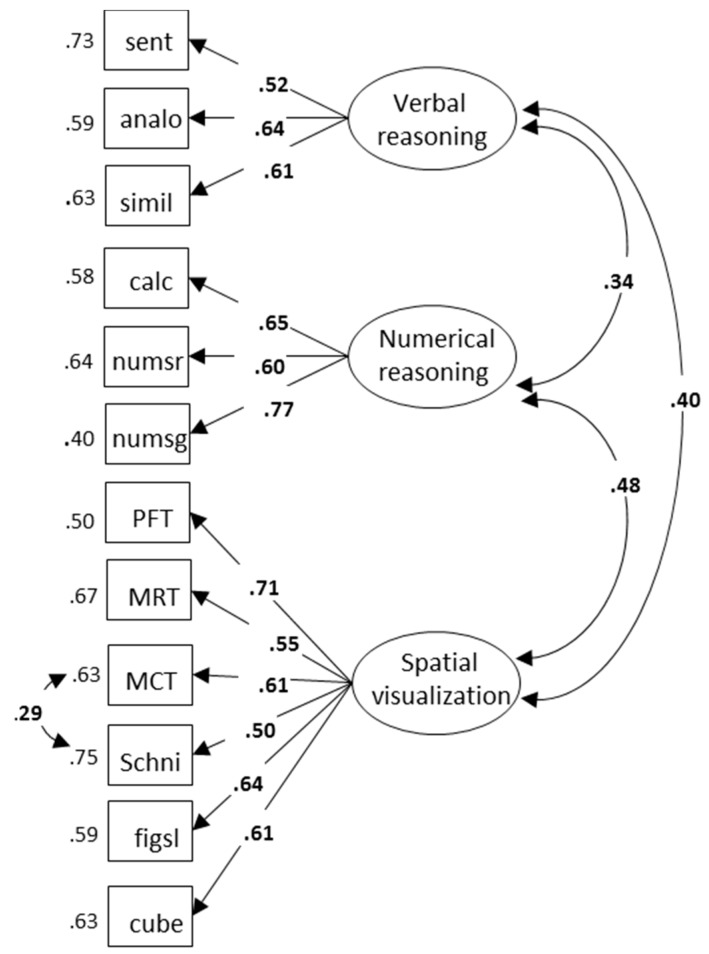
Three correlated factors model of cognitive abilities. Sent = Sentence completion; Analo = Analogies; Simil = similarities; Calc = Calculations; Numsr = Number series; Numsg = Numerical signs; PFT = Paper Folding Test; MRT = Mental Rotations Test; MCT = Mental Cutting Test; Schni = Schnitte; Figsl = Figure selection. All paths are significant at *p* < 0.001.

Prior to estimating the relations between abilities and academic achievements, we conducted exploratory and confirmatory factor analyses in order to establish a model of cognitive abilities. The detailed analyses can be found in the supplementary materials (Section 2). Here, we report the final model that was retained. The respective correlation matrix of observed variables is presented in Table 4 and the model is shown in Figure 1. Fit statistics for this model were as follows: χ^2^/df = 95.42/50, *p* ≤ 0.001; RMSEA (90% CI) = 0.05 (0.04–0.07); CFI = 0.94; SRMR = 0.05. In this model, the three verbal tests were indicators of a verbal reasoning factor; the three numerical tests were indicators of a numerical reasoning factor; and the six SV tests were indicators of an SV factor. Based on prior analyses, the residual variances of the two cross-sectioning tests were freely estimated. Tests for measurement invariance of this model across the groups are reported in the supplementary materials (Section 3).

### 3.6. Prediction of Academic Achievements

Table 5 and Table 6 present the observed correlations between cognitive measures and grades among engineering and math-physics students respectively (the full correlation matrices are included in the supplementary materials Section 1). It is evident from these tables that SV tests significantly correlated with grades on two engineering courses (machine elements and technical drawing and Computer Aided Design (CAD)), whereas none correlated significantly with math and with mechanics among engineering students. It is also evident that numerical ability tests significantly correlated with all of the grades in this group. Among math-physics students, all ability measures significantly correlated with almost all of the grades. Notably, of the SV tests, only MCT and Schnitte—the two cross-sectioning tests—significantly correlated with grades.

Table 7 and Table 8 present correlations between latent abilities and grades in each group respectively. Course grades were retained as observed variables in order to keep a differentiation between the courses.^1^ The correlations in Table 7 and Table 8 indicate how each of the abilities related to grades *without* controlling for other abilities (i.e., bidirectional paths in a measurement model). As before, numerical reasoning was the primary factor related to math and mechanics grades among engineering students, whereas SV was related only to two ‘spatial’ courses; among math-physics students, all abilities were correlated with all of the grades.

**Figure 2:**
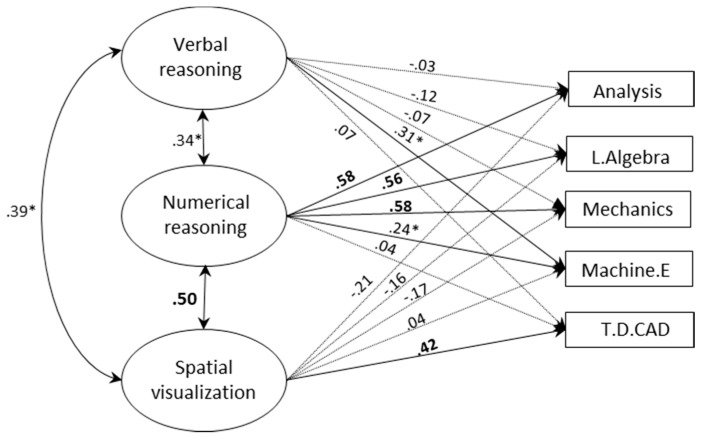
A SEM with three correlated factors for predicting grades among engineering students (*N* = 150). Bolded values indicate *p* < 0.001; * *p* < 0.05.

**Figure 3:**
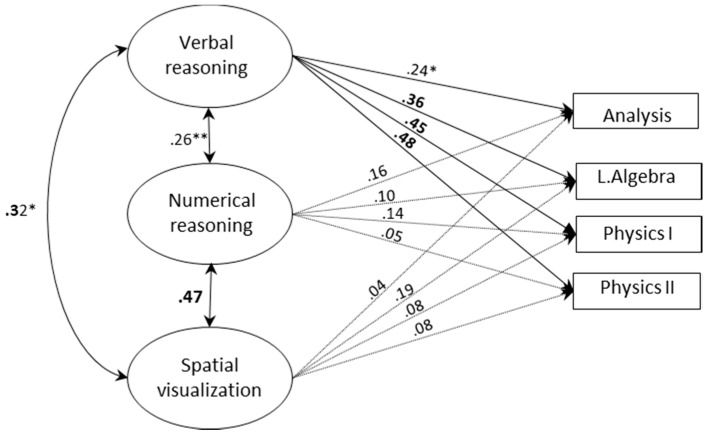
A SEM with three correlated factors for predicting grades among math-physics students (*N* = 167). Bolded values indicate *p* < 0.001; ** *p* < 0.01; * *p* < 0.05.

Figure 2 and Figure 3 display the structural models with three correlated factors for each of the study groups respectively (fit statistics are presented in Table 9). In these models, the effects of the three abilities on grades were simultaneously estimated. As in multiple regression, each of the effects indicates the degree to which an ability predicts grades over and above the other abilities in the model (further analyses confirmed that removing non-significant paths did not result in worse fit, confirming these paths were redundant).

As shown in Figure 2 and Figure 3, the pattern of prediction differed between study programs, ability domains and areas of achievements. SV remained a significant predictor of grades on a highly ‘spatial’ course in the engineering program (T.D.CAD). The effect of SV on the other engineering course (machine elements) was no longer significant. Among math-physics students, SV had no incremental predictive power for any of the grades. Thus, SV did not contribute beyond other abilities to grades on math-intensive courses. Verbal and numerical abilities were also differentially related to achievements in each group. Among engineering students, numerical ability had strong unique effects on math and physics courses, whereas among math-physics students its effects were no longer significant once other abilities were controlled for. Verbal ability, in turn, had unique effects on all grades among math-physics students, but not among engineering students (except for the course machine elements).

**Figure 4:**
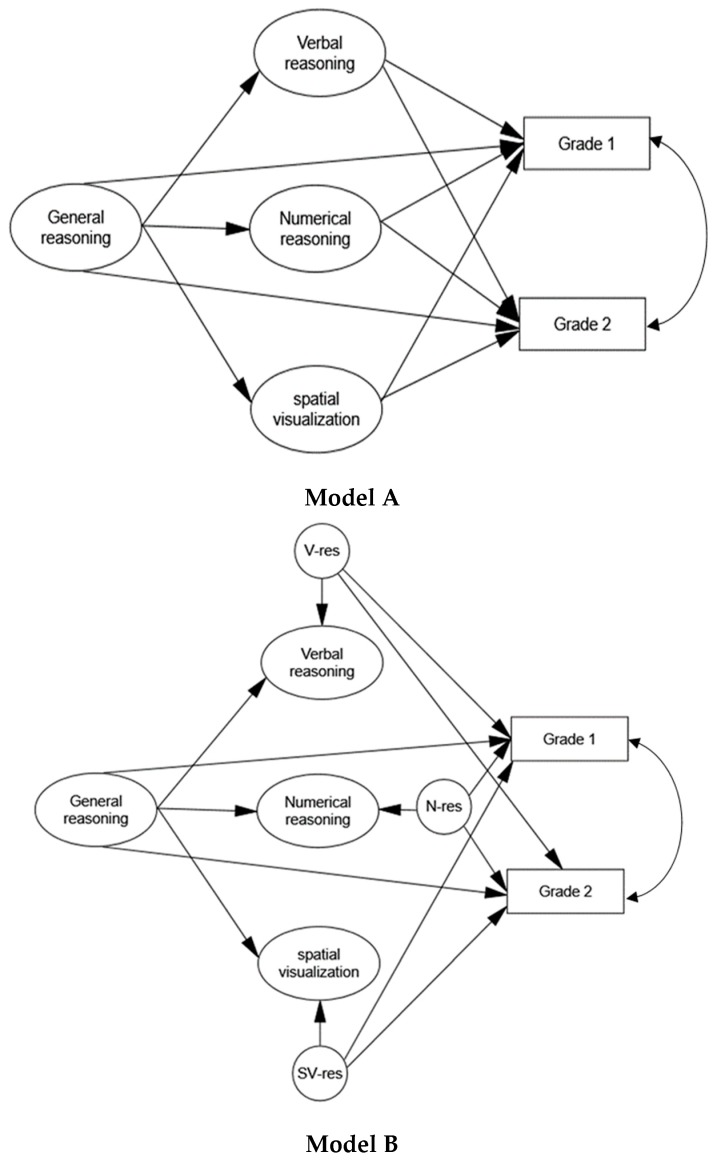
(**Top**) Model for predicting grades with general and specific abilities. (**Bottom**) Model for predicting grades with a general factor and the residuals of specific abilities. V-res = residual of verbal reasoning; N-res = residual of numerical reasoning; SV-res = residual of spatial visualization.

The contribution of domain-general factors to achievement prediction was indirectly inferred from the above models. That is, if initially a factor had significant correlations with grades (see Table 7 and Table 8), but in the structural model it had no significant effects on these grades, then the initial correlations were assumed to be due to some commonality between the ability factors (hence ‘domain general’). To disentangle the effects of specific abilities from domain-general factors, we additionally tested models in which a general ability factor was included. Thus, we replaced factors-correlations with a second-order factor that had direct paths to each of the specific abilities. Conceptually, we assumed that this factor captured reasoning ability that is needed for performance in the three ability domains. We then performed two types of analyses. In the first, we estimated the effects of both first- and second-order factors on grades (model A in Figure 4). In the second, we estimated the effects of the residual variances of the first-order factors as well as of the general factor on grades (model B in Figure 4). The residual terms of the first-order factors represent the part of the variance in these factors that is not explained by the general factor. The second method should therefore indicate more precisely whether specific abilities are predictive of the outcomes when the effects of the general factor are completely controlled for (e.g., [66]).

An initial estimation of all paths simultaneously (i.e., paths from all of the specific factors, or their residuals, and from the general factor to the outcome variables) yielded identification problems, presumably because many paths were redundant and had estimates close to zero. We therefore prespecified the models, removing paths with negative and close to zero estimates. The standardized path coefficients for the resulting models are presented in Table 9 and model fit in Table 10.

Overall, the pattern of effects in models A and B was largely similar to the one in the correlated factors model. That is, most of the effects of specific abilities remained significant beyond a general ability factor. However, when specific effects were estimated from the residual variances of the specific abilities (as in model B), some of these effects became weaker, and the direct effects of the general factor became stronger. This was the case for grades on the two engineering courses *machine elements* and *T.D.CAD*, and for all of the grades in the math-physics group. In contrast, the specific effects of numerical reasoning on math and physics (mechanics) in the engineering group remained consistently strong across the models. Taken together, isolating domain-specific from domain-general effects still showed strong domain-specific effects in both of the groups, but in some cases domain-general factors clearly contributed to the prediction. Considering the initially significant correlations between all ability factors and grades among math-physics students (see Table 8), the stronger domain-general effects in this group could be expected. It should be noted that the overlap between specific abilities in our sample was likely lower than in more heterogeneous samples (this is often found in high-ability groups [67]), possibly limiting its predictive power.

## 4. Discussion

While previous research showed the unique predictive validity of early-assessed SA for long-term STEM achievements, we investigated whether ability differences among students who entered higher STEM education can be linked with their achievements during the first undergraduate year. Our primary focus was on the particular role of SV ability for different STEM topics. Additionally, as we were dealing with a high-ability sample, our investigation also challenged the view that cognitive abilities forfeit their predictive power in highly selected samples. Differently from other studies on advanced STEM learning, we systematically differentiated between ability domains, domains of achievements, study programs and ability measures. Overall, our data clearly showed that ability differences in this high-ability population are relevant to achievement prediction, corroborating previous findings against a ‘threshold’ view of cognitive abilities [34,44,48,49]. The results were also clear with respect to our two research questions, namely whether SV predicts achievements on STEM courses beyond numerical and verbal reasoning abilities, and whether SV differs in its predictive validity between domains of achievements. Across ability measures and study programs, SV did not uniquely predict achievements in math-intensive courses, which constitute a major part of the curriculum. In fact, SV was unrelated to the math and physics grades of engineering students even before controlling for other abilities. Among math-physics students, two cross-sectioning tests significantly correlated with math and physics grades, but without incremental effects over other abilities or a domain-general ability factor. In contrast, SV had unique predictive validity for achievements on an engineering technical drawing course. Moreover, numerical and verbal reasoning abilities were uniquely predictive of achievements on most math and physics courses. Thus, the predictions seem highly domain specific: differences in SV, even among the spatially talented, mattered when complex spatial tasks were in focus (technical drawing), whereas differences in numerical or verbal reasoning abilities among the mathematically talented mattered when math was in focus. Nonetheless, at least for math-physics students, the effects on math and physics grades were partly driven by domain-general factors. Moreover, when a general factor was controlled for, the effect of SV on technical drawing grades decreased. It therefore appears that rather than broadly determining STEM success during the novice phase, as would be expected based on prior research assumptions, SV emerged as a narrower STEM predictor relative to other abilities, which in turn predicted success on a broader range of core STEM subjects. Apparently, when it comes to predicting mathematics achievements, SV seems to have a lower threshold than numerical and verbal abilities (i.e., a lower level is sufficient for success). We next discuss further explanations and implications of these results.

### 4.1. Predictions at a High-Ability Range 

One may suspect that the lack of correlations between SV and math-based achievements is a result of a restriction of range in SV, and that in more heterogeneous samples positive correlations would emerge. We cannot rule out this possibility. However, our goal was to study predictors of achievements among students who select advanced STEM programs rather than among students in general. The different patterns of abilities–achievements relations that we found indicate that in spite of the high-ability range in this group, sufficient variability existed for detecting effects. For this reason, we find it unlikely that the weak correlations between SV and math achievements found here are entirely due to a restriction of range, but rather assume they indicate a weaker relevance of SV to some domains of achievements. It is also noteworthy that the highly challenging test ‘Schnitte’, which was specifically designed for individuals with high SA, yielded the same pattern of links with grades as the other tests, even though its score range was broader.

Nonetheless, to elaborate on the more general case, it should be noted that all of the abilities were at the high range in this sample, and numerical ability even more so than SV. Consequently, all of the effects are potentially underestimated if generalizations to a broader population are to be made. The effects in a less selective sample are thus expected to be magnified proportionally: numerical and verbal abilities will still have stronger effects on math than SV. If indeed a higher frequency of lower SV scores were necessary to find effects on math achievements, one possible implication could be that poor SV ability is a stronger marker than exceptional SV ability for succeeding or not in advanced math learning. This would, in fact, be in line with findings on SV–math relations among students who performed poorly on SV tests (e.g., [35]). Finally, to the extent that SV ability predicts STEM achievements more strongly in a lower ability range, it would remain to be determined whether this stems from spatial-visual factors or from domain-general ones. The point may even be more important in less selective samples, because the overlap between cognitive abilities (i.e., domain generality) tends to be stronger in lower ability ranges [67]. With higher variability in general ability, its contribution to achievement prediction is likely to be stronger. 

### 4.2. Correlates of Advanced Mathematics

It appears that for students who are not particularly poor in SV, being more talented spatially might not further contribute to achievements in math-intensive courses, at least in those that form the basis for even more advanced courses. That SV nonetheless predicted technical drawing grades in this sample suggests that the courses substantially differ in their spatial demands. It is possible that advanced math courses, although highly demanding, do not draw on exceptional spatial skills as more specialized engineering courses do. Math courses may focus on translating complex spatial structures into abstract mathematical formalism, more than they require superb visualization of such structures. Furthermore, ‘space’ in advanced math is often not entirely familiar from “real world” experience, for example when dealing with dimensions higher than three. Although students may use mental images to visualize abstract math concepts, these do not necessarily require particularly high spatial skills, nor is it clear whether they are crucial for better learning. This does not mean that SV ability is unimportant to advance math, but rather points at a possible limitation of SV as a cognitive resource for learning higher math. Additionally, our finding that positive correlations between one type of SV tests (visualization of cross-sections) and some math achievements were mediated by factors common to other domains of reasoning suggests that factors other than visualization—which may be strongly present in some spatial tests—play a role when it comes to mathematics.

The results additionally suggest differential cognitive demands between math-intensive courses, since cognitive abilities related differently to math-courses across study programs. As mentioned in the method section, mathematics is taught in essentially different ways in each of the study programs we investigated. An informal inquiry among math faculty (who teach these courses in both programs) informed us that for math-physics students, math is typically highly theoretical and centrally dealing with proof learning. It has also been described as requiring an extreme change in the ways in which students are used to thinking of mathematics at the Gymnasium, as it introduces concepts that sometimes contradict their previously acquired knowledge and intuitions. Mathematics for mechanical engineers, although also highly abstract compared with Gymnasium classes, is typically more strongly linked with real world applications and relies more often on calculations than on proof. Although merely descriptive, we speculate that such differences in teaching approach have implications on the kinds of mental processes and abilities that are required for successful learning. For example, application-oriented mathematics may require high efficiency in utilizing domain-specific skills and knowledge, which may explain the almost exclusive relation between numerical reasoning ability and math in the engineering group. Math that is more theoretical and proof-based may require additional reasoning abilities and rely less critically on calculation efficiency. The stronger domain-general links of abilities-grades in the math-physics group and the unique link with verbal reasoning may be seen as indications in this direction. Obviously, a more systematic investigation would be needed in order to confirm these observations.^2^ Nevertheless, the data provides initial evidence that even within the same mathematical areas (i.e., calculus and algebra) basic cognitive abilities can have different roles. 

### 4.3. The Present Results in Light of Previous Research

Our results seem inconsistent with findings on the unique predictive validity of SA among students of extremely high ability (e.g., the top 0.01% in Lubinski and Benbow, 2006). There are some obvious differences between the present study and these earlier ones, primarily in scale, the kinds of outcomes considered, and more broadly being conducted in educational systems that may substantially differ. Such factors likely limit the comparability of results. Nevertheless, we assume some invariance in the cognitive aspects of STEM learning across social-cultural systems, and find additional explanations noteworthy. First, the timing of ability assessment might be critical, as ability differences during early adolescence are likely to be influenced by ongoing cognitive development. Thus, differences in earlier age might reflect not only ability level per se, but also, for example, differences in developmental rates. These may considerably influence the prediction of future outcomes. Additionally, the studies on gifted adolescents used above-level testing (i.e., tests that were designed for college admission rather than for 13-year olds), which ensured large score ranges within a highly selected group. It is plausible that using tests that are more challenging in our sample would have yielded different results, though it is difficult to predict in what way. In particular, it is unclear whether higher variance further up the SV scale (i.e., due to more difficult tests), would have revealed effects where variance in lower levels did not. Also noteworthy is that in predicting long-term achievements, early ability differences may be markers of general potential for learning and creativity, more than they indicate which abilities are engaged in particular learning tasks. Moreover, while the above studies predicted general achievement criteria (e.g., choices, degrees, publications), we focused on the interplay between abilities and specific achievements, which we assumed more closely indicate which abilities are relevant to STEM learning. The differences in prediction patterns may have therefore stemmed, in part, from different choices of outcome criteria, and in this respect should be seen as complementary. Finally, measures of SA in previous studies might have involved more non-spatial factors than in the present one, and these might have influenced predictions. For example, in Wai et al. (2009), SA included a figural matrices test, which is a strong indicator of fluid reasoning, and a mechanical reasoning test, which is highly influenced by prior experience with mechanical rules. These factors may, in themselves, be essentially important to STEM, but they do not necessarily reflect SV ability.

Studies that examined SA and advanced mathematics among STEM students are scarce. Miller and Halpern [44] found positive effects of SA training on physics but not math grades of high-ability STEM students, and concluded that SA training was not relevant to the content of math courses, which is consistent with our own conclusion. Some studies with younger populations also found small or no effects of SA on math performance [3], but others found more positive relations between SA and math, mostly among non-STEM students or with simpler forms of math [35,64]. Further research on the role of SA in advanced math learning therefore seems warranted. 

Finally, we were surprised by the weak correlations between SV and physics grades in both of the groups, and particularly with mechanics, which has been positively related to SV before [10,68]. One possible explanation for these differences is that students in our sample may have had higher levels of prior-knowledge in science: first at entry level due to self-selection, and at the time of achievements assessment (i.e., course exams), as it reflected learning during an entire academic year. As suggested by Uttal and Cohen [16], SA may be particularly important when domain-knowledge is low. Consistent with this, SV was related to mechanics problem solving [10,68] and to advanced math problems [36] among non-STEM students who had no formal knowledge on these topics. It follows that if students who select STEM programs have enough prior knowledge so that SV ability is not ‘needed’ for many topics, then the connection between SV ability and STEM is more relevant before rather than after entry into undergraduate STEM. This is, in fact, consistent with findings that SA was particularly predictive of later choice in STEM comparing to other domains [4]. Nonetheless, students in our sample can by no means be regarded ‘experts’. Although they may have acquired STEM knowledge in high school, they are novices with respect to many new and challenging knowledge domains at the undergraduate level. 

## 5. Conclusions

The results of this study highlight the importance of differentiating between domains of ability as well as domains of achievements in research on SA and STEM. Additionally, a distinction between the predictive power of early SA and its role in later STEM learning may be useful. When it comes to advanced STEM learning, different thresholds may exist for different disciplines. While SA may play a role in advanced math learning, it might not be as critical as other reasoning abilities, or as it is for spatially demanding topics. Considering that math-based courses are at the core of STEM education and critical particularly to beginners, this has potential implications on the way in which students are prepared for higher education in STEM. For example, SA training might benefit performance in particular spatial tasks, but may not be the optimal way of improving students’ achievements in math-intensive courses. Even if SA training yields learning gains in math in less selected samples, these could be driven by factors not specific to visualization. Understanding what is, in fact, being trained in SA training could help clarify these questions.

The point may also be important with respect to the ongoing discussion on women’s underrepresentation in STEM (e.g., [69]). Although it is beyond the scope of this study to directly address this issue, the possibility that high spatial skills are not crucial for higher mathematics puts in a different light the often-made arguments linking women’s poorer performance in SA tests to this gender gap. Interestingly, even the highly competent women in our study tended to do worse than men in mental rotations, but there was no indication that women had less success in their math-intensive courses, and mental rotation was unrelated to math achievements across groups. Nonetheless, since the number of women in our sample was too small for drawing strong conclusions, we think that future research is needed in order to clarify whether the current results apply to high-ability women in advanced STEM education. 

### Limitations

The present study was not a large-scale one, therefore generalizations to the population of STEM undergraduates are yet to be shown. Nonetheless, the results were consistent across cohorts, study programs and ability measures, providing grounds to further investigate these issues in larger samples and comparable universities. Ideally, using tests that are more challenging in these populations would yield larger ability differences and thus enable further exploration of their impact. Additionally, our frequent reference to studies conducted in U.S. populations raises factors such as differences in educational systems and even cultural differences as potential limiting factors for the generalizability of the results. For example, in the U.S., students choose their major at a later point in their undergraduate studies comparing to Europe and other parts of the world, where they enroll on a particular program from the start. Thus, it is difficult to directly compare the category ‘STEM beginners’ between these systems. We nevertheless assume that STEM programs worldwide are similar in several basic aspects, such as the centrality of mathematics, hence the present findings should be relevant beyond educational systems. Finally, we focused on two major STEM disciplines, but this is still only a partial selection out of the variety of disciplines in this category. Similarly, although our outcome variables represent major areas of study during the first undergraduate year, they are not an exhaustive selection of achievements. Research on other domains of STEM, on further types of achievements as well as in more advanced years, could further inform our knowledge on the particular role of SA in advanced STEM learning.

## Figures and Tables

**Table 1:** Participation rates by sample, field and gender.

Cohort	N	Engineering	Math-Physics
2012 (pilot)	65	38	27
2013	158	72	86
2014	94	40	54
Total	317	150	167
Women (%)	44 (14%)	14 (9%)	30 (18%)

**Table 2:** Means, standard deviations and reliability estimates of all measures included in the study.

Ability	Test	Mean	*SD*	Scale Range	Skew	Reliability	
						Cronbach’s Alpha ^a^	GLB
Spatial visualization	Paper folding	16.71	2.46	0–20	−0.41	0.73	0.85
	M.Rotations	16.10	3.96	0–24	−0.19	0.80	0.86
	Mental cutting	19.20	3.88	0–25	−0.63	0.78	0.87
	Schnitte	8.55	3.04	0–17	−0.08	0.61	0.73
IST: Figural reasoning	Figure selection	14.06	3.39	0–20	−0.65	0.72 (0.76)	0.84
	Cubes	14.56	3.66	0–20	−0.55	0.79 (0.79)	0.86
	Matrices	11.62	2.73	0–20	−0.14	0.60 (0.66)	0.75
**Total figural**		40.24	7.06	0–60	−0.31	0.85 (0.87)	0.81
IST: Verbal reasoning	S.completion	14.21	2.74	0–20	−0.73	0.56 (0.63)	0.72
	Analogies	14.24	2.12	0–20	−0.24	0.36 (0.68)	0.58
	Similarities	12.61	2.55	0–20	−0.37	0.54 (0.71)	0.67
**Total verbal**		41.06	5.54	0–60	–0.45	0.70 (0.88)	0.88
IST: Numerical reasoning	Calculations	17.03	2.48	0–20	−0.92	0.70 (0.80)	0.78
	Number series	17.21	2.51	0–20	−1.46	0.77 (0.90)	0.85
	Numerical signs	17.24	2.52	0–20	−0.77	0.71 (0.84)	0.82
**Total numerical**		51.48	5.84	0–60	−0.85	0.85 (0.95)	0.85

M.Rotations = Mental rotations; S.completion = sentence completion; GLB = greatest lower bound; ^a^ For IST sub-scales: values in brackets are based on a normative sample of high-school graduates (*N* = 1445); for IST sum-scores, values in brackets are based on a total (mixed) normative sample (*N* = 3484).

**Table 3:** Means and standard deviations (in parentheses) of raw sum-scores on the IST in the present sample and in two normative samples.

Ability	Current Sample	Normative Sample	
		High-School Graduates ^a^	General Population ^b^
Verbal	41.06 (5.54)	36.94 (8.34)	31.56 (7.89)
Numerical	51.48 (5.84)	39.19 (12.19)	33.04 (11.25)
Figural	40.24 (7.06)	33.30 (8.34)	30.84 (8.50)
General	132.39 (13.26)	107.63 (21.24)	95.17 (22.59)

^a^ 202 high-school graduates from Germany (age group 19–20); ^b^ 1190 German participants with various educational backgrounds (ages 15–20). Norms were established in the year 2000.

**Table 4:** Correlations between scores on ability tests (*N* = 317).

Test	1	2	3	4	5	6	7	8	9	10	11	12	13
1. PFT	-												
2. MRT	0.37 ***	-											
3. MCT	0.40 ***	0.36 ***	-										
4. Schnitte	0.37 ***	0.23 **	0.50 ***	-									
5. Figsl	0.47 ***	0.35 ***	0.41 ***	0.37 ***	-								
6. Cube	0.46 ***	0.40 ***	0.35 ***	0.22 ***	0.34 ***	-							
7. Sent	0.12	−0.03	0.19 **	0.21 ***	0.08	0.06	0.04	-					
8. Analo	0.27 ***	0.08	0.23 **	0.32 ***	0.16 **	0.09	0.15 *	0.33 ***	-				
9. Simil	0.22 **	0.05	0.12	0.25 ***	0.12 *	0.10	0.13	0.34 ***	0.37 ***	-			
10. Calc	0.15 *	0.12	0.26 ***	0.16 *	0.20 ***	0.23 ***	0.14	0.16 **	0.16 **	0.13 *	-		
11. Numsr	0.19 *	0.15	0.18 **	0.08	0.13	0.21 ***	0.23 ***	0.01	0.16 **	0.13	0.40 ***	-	
12. Numsg	0.21 ***	0.29 ***	0.22 ***	0.19 ***	0.24 ***	0.32 ***	0.24 ***	0.06	0.18 ***	0.20 *	0.49 ***	0.46 ***	-

PFT = Paper Folding Test; MRT = Mental Rotations Test; MCT = Mental Cutting Test; Figsl = Figure selection; Sent = Sentence completion; Analo = Analogies; Simil = similarities; Calc = Calculations; Numsr = Number series; Numsg = Numerical signs. * *p* < 0.05, ** *p* < 0.01, *** *p* < 0.001.

**Table 5:** Correlations between ability measures and grades among engineering students (*N* = 150).

AbilityT	Test	Analysis	L.Algebra	Physics (Mechanics)	Machine Elements	T.D.CAD
Spatial visualization	Paper Folding	0.07	0.09	0.11	0.25 **	0.35 ***
	Mental Rotation	−0.03	−0.04	0.00	0.15	0.29 ***
	Mental Cutting	0.14	0.15	0.06	0.25 *	0.33 ***
	Schnitte	0.10	0.07	0.06	0.16	0.30 ***
	Figure selection	0.03	0.04	0.06	0.20 *	0.28 **
	Cubes	0.00	0.02	0.03	0.07	0.23 **
Verbal reasoning	S.completion	0.04	−0.02	−0.01	0.26 ***	0.05
	Analogies	0.14	0.14	0.18	0.32 ***	0.27 **
	Similarities	−0.04	−0.10	−0.07	0.11	0.14
Numerical reasoning	Calculations	0.39 ***	0.32 ***	0.37 ***	0.26 **	0.24 **
	Num. series	0.26 **	0.27 **	0.30 **	0.27 **	0.09
	Num. signs	0.20 *	0.24 **	0.23 **	0.18 *	0.14

T.D.CAD = technical drawing and CAD; * *p* < 0.05, ** *p* < 0.01, *** *p* < 0.001.

**Table 6:** Correlations between ability measures and grades among math-physics students (*N* = 167).

Ability	Test	Analysis	L.Algebra	Physics I (Mechanics)	Physics II (Electricity)
Spatial visualization	Paper Folding	0.16	0.25 *	0.18 *	0.16
	Mental Rotation	0.06	0.15	0.12	0.06
	Mental Cutting	0.24 **	0.34 ***	0.35 ***	0.35 ***
	Schnitte	0.10	0.27 **	0.25 **	0.30 ***
	Figure selection	0.09	0.18 *	0.13	0.11
	Cubes	0.02	0.10	0.03	0.00
Verbal reasoning	S.completion	0.18	0.25 **	0.39 ***	0.42 ***
	Analogies	0.20 *	0.25 **	0.27 **	0.28 ***
	Similarities	0.10	0.29 ***	0.21 *	0.19 *
Numerical reasoning	Calculations	0.28 **	0.34 ***	0.34 ***	0.28 ***
	Num. series	0.10	0.10	0.05	0.02
	Num. signs	0.20 *	0.19 *	0.25 *	0.17

* *p* < 0.05, ** *p* < 0.01, *** *p* < 0.001.

**Table 7:** Correlations between latent abilities and grades among engineering students.

Title	Analysis	L.Algebra	Physics (Mechanics)	Machine Elements	T.D.CAD
Spatial visualization	0.07	0.08	0.10	0.27 **	0.46 ***
Verbal reasoning	0.08	0.01	0.06	0.40 ***	0.24
Numerical reasoning	0.46 ***	0.44 ***	0.48 ***	0.36 ***	0.27 *

* *p* < 0.05, ** *p* < 0.01, *** *p* < 0.001.

**Table 8:** Correlations between latent abilities and grades among math-physics students.

Latent ability	Analysis	L.Algebra	Physics I	Physics II
Spatial visualization	0.19	0.35 ***	0.29 **	0.26 **
Verbal reasoning	0.29 **	0.45 ***	0.51 ***	0.52 ***
Numerical reasoning	0.24 *	0.28 **	0.29 **	0.21 *

* *p* < 0.05, ** *p* < 0.01, *** *p* < 0.001; T.D.CAD = technical drawing and CAD.

**Table 9:** Standardized path coefficients for models A and B in each group.

Course Grades	Model A General and Specific Abilities				Model B General and Specific Residuals			
	V	N	SV	g	V	N	SV	g
Engineering								
Analysis	-	0.43 ***	-	-	-	0.51 **	-	0.14
Linear algebra	-	0.41 ***	-	-	-	0.51 **	-	0.12
Mechanics	-	0.45 ***	-	-	-	0.51 **	-	0.16
Machine.E	0.27 *	-	-	0.34 *	0.19	-	-	0.45 ***
T.D.CAD	0.11	-	0.46 ***	-	-	-	0.29 **	0.38 **
Math-physics								
Analysis	0.25 *	0.16	-	-	0.18	-	-	0.29 *
Linear algebra	0.32 **	-	-	0.31 *	0.28 *	-	-	0.46 **
Physics I	0.42 **	-	-	0.22	0.37 **	-	-	0.41 **
Physics II	0.48 **	-	-	0.12	0.42 **	-	-	0.34 **

V = verbal reasoning; *N* = Numerical reasoning; SV = spatial visualization; g = general ability; Cells without values are path estimates that were removed due to close to zero or negative values; *** *p* < 0.001; ** *p* < 0.01; * *p* < 0.05.

**Table 10:** Fit statistics for models A and B in each group.

Model	χ^2^	df (*p*)	RMSEA (90% CI)	CFI	SRMR
Engineering					
Model A	134.62	103 (0.02)	0.05 (0.02–0.07)	0.962	0.07
Model B	133.05	100 (0.02)	0.05 (0.02–0.07)	0.960	0.06
Math-physics					
Model A	138.92	90 (<0.001)	0.06 (0.04–0.08)	0.944	0.07
Model B	140.56	90 (<0.001)	0.06 (0.04–0.08)	0.942	0.07

## Footnotes

1 Since math-based grades were highly correlated, we estimated in subsequent analysis a similar model with a latent variable for math, which strengthened the current pattern of results.

2 The above discussion builds on the common assumption that cognitive abilities relate to various outcomes because they serve as a cognitive resource, i.e., that some causality is involved. Obviously, we cannot test this assumption directly here.

## Supplementary Materials

The following are available online at http://www.mdpi.com/2079-3200/6/4/48/s1, Table S1: Observed correlations between abilities and grades among mechanical engineering students (N = 150); Table S2: Observed correlations between abilities and grades among math-physics students (N = 167); Table S3: Factor loadings for a 2-factors solution in an oblique EFA; Table S4: Multiple group analysis for measurement invariance; Table S5: Correlations between ability measures and grades among engineering students (N = 133); Table S6: Correlations between ability measures and grades among math-physics students (N = 140); Table S7: Fit statistics for measurement models in each group; Figure: S1: CFA on SV tests; Figure S2: A SEM for predicting grades among engineering students (N = 133); Figure S3: A SEM for predicting grades among math-physics students (N = 140).
