# Supplementary material for: Which Cognitive Abilities Make the Difference? Predicting Academic Achievements in Advanced STEM Studies
Source: J Intell. 2018 Oct 26;6(4):48. doi: 10.3390/jintelligence6040048 (PMC6480791; doi:10.3390/jintelligence6040048)
Supplement: Supplementary file 1 [file jintelligence-06-00048-s001.pdf]

Journal of Intelligence

MDPI

Supplementary Materials

Which Cognitive Abilities Make the Difference? Predicting Academic Achievements in Advanced STEM Studies

Michal Berkowitz * and Elsbeth Stern

I. Correlations in Separate Groups

Table S1. Observed correlations between abilities and grades among mechanical engineering students (N = 150).

	SV tests						Verbal tests			Numerical tests			Grades				
	1	2	3	4	5	6	7	8	9	10	11	12	13	14	15	16	17
1. PFT	-																
2. MRT	0.45	-															
3. MCT	0.40	0.34	-														
4. Schnitte	0.37	0.30	0.45	-													
5. Figsl	0.48	0.32	0.41	0.38	-												
6. Cube	0.50	0.54	0.32	0.26	0.28	-											
7. Sent	0.10	−0.02	0.22	0.18	0.11	−0.01	-										
8. Analo	0.33	0.19	0.27	0.30	0.26	0.17	0.33	-									
9. Simil	0.13	0.14	0.09	0.13	0.11	0.16	0.38	0.26	-								
10. Calc	0.16	0.17	0.25	0.19	0.21	0.32	0.19	0.16	0.11	-							
11. Numsr	0.19	0.19	0.14	0.07	0.03	0.16	0.02	0.17	0.11	0.35	-						
12. Numsg	0.24	0.29	0.16	0.18	0.18	0.32	0.02	0.15	0.21	0.45	0.36	-					
13. Analysis	0.07	−0.03	0.14	0.10	0.03	0.00	0.04	0.14	−0.04	0.39	0.26	0.20	-				
14. Linalg	0.09	−0.04	0.15	0.07	0.04	0.02	−0.02	0.14	−0.10	0.32	0.27	0.24	0.79	-			
15. Mech	0.11	0.00	0.06	0.06	0.06	0.03	−0.01	0.18	−0.07	0.37	0.30	0.23	0.79	0.79	-		
16. Machel.	0.25	0.15	0.25	0.16	0.20	0.07	0.26	0.32	0.11	0.26	0.27	0.18	0.59	0.59	0.61	-	
17. T.D.CAD	0.35	0.29	0.33	0.30	0.28	0.23	0.05	0.27	0.14	0.24	0.09	0.14	0.34	0.42	0.44	.50	-
Mean	16.46	16.29	18.92	8.00	14.02	14.10	14.13	13.91	12.07	16.68	16.93	16.89	4.05	4.11	4.50	4.50	5.20
SD	2.60	3.82	3.96	2.76	3.51	3.55	2.70	1.94	2.60	2.39	2.72	2.54	1.25	1.20	0.96	.67	.42

PFT=Paper Folding Test; MRT=Mental Rotations Test; MCT=Mental Cutting Test; Figsl=Figure selection; Sent=Sentence completion; Analo=Analogies; Simil=similarities; Calc=Calculations; Numsr=Number series; Numsg=Numerical signs; Linalg=Linear Algebra; Mech=Mechanics; Machel=Machine elements; T.D.CAD=Technical Drawing and CAD.

Table S2. Observed correlations between abilities and grades among math-physics students (N = 167).

	SV tests						Verbal tests			Numerical tests			Grades			
	1	2	3	4	5	6	7	8	9	10	11	12	13	14	15	16
1. PFT	-															
2. MRT	0.30	-														
3. MCT	0.40	0.39	-													
4. Schnitte	0.37	0.20	0.54	-												
5. Figsl	0.47	0.37	0.42	0.37	-											
6. Cube	0.40	0.31	0.37	0.16	0.40	-										
7. Sent	0.15	−0.03	0.16	0.24	0.06	0.11	-									
8. Analo	0.20	0.00	0.19	0.30	0.08	0.01	0.33	-								
9. Simil	0.28	−0.00	0.13	0.31	0.15	0.00	0.31	0.44	-							
10. Calc	0.11	0.08	0.25	0.09	0.18	0.13	0.13	0.13	0.09	-						
11. Numsr	0.17	0.13	0.22	0.07	0.24	0.24	−0.01	0.13	0.13	0.43	-					
12. Numsg	0.17	0.30	0.27	0.16	0.30	0.29	0.09	0.17	0.14	0.52	0.54	-				
13. Analysis	0.16	0.06	0.24	0.10	0.09	0.02	0.18	0.20	0.10	0.28	0.10	0.20	-			
14. Linalg	0.25	0.15	0.34	0.27	0.18	0.10	0.25	0.25	0.29	0.34	0.10	0.19	0.73	-		
15. PhysI	0.18	0.12	0.35	0.25	0.13	0.03	0.39	0.27	0.21	0.34	0.05	0.25	0.70	0.75	-	
16. PhysII	0.16	0.06	0.35	0.30	0.11	0.00	0.42	0.28	0.19	0.28	0.02	0.17	0.71	0.75	0.82	-
Mean	16.94	15.94	19.46	9.00	14.06	14.96	14.28	14.56	13.09	17.36	17.47	17.56	3.86	3.97	4.49	4.07
SD	2.31	4.06	3.79	3.19	3.26	3.69	2.77	2.22	2.41	2.51	2.72	2.48	1.08	1.16	.88	1.08

PFT=Paper Folding Test; MRT=Mental Rotations Test; MCT=Mental Cutting Test; Figsl=Figure selection; Sent=Sentence completion; Analo=Analogies; Simil=similarities; Calc=Calculations; Numsr=Number series; Numsg=Numerical signs; Linalg=Linear Algebra; Mech=Mechanics; PhysI=Physics I; PhysII=Physics II.

Journal of
Intelligence

II. Model Specification

Spatial Visualization (SV)

There were six tests we expected to load on a SV factor: Paper folding, Mental rotations, Mental cutting (MCT), Schnitte, Figure-selection and Cube. Since these tests were not developed within a single test-battery, we first conducted an exploratory factor analysis (EFA; geomin oblique rotation) on these tests only. This indicated that a two factors solution fitted the data significantly better than a one factor solution (χ^2 /df = 26.05/5, $p < 0.001$). Factor loadings for the two-factor solutions are shown in Table S3. The two cross-sectioning tests (Schnitte and MCT) loaded on a factor which was distinct from the one explaining variance in the rest of the tests. The MCT had significant loadings on both factors. Thus, the different SV tests were not as unitary as expected.

Table S3. Factor loadings for a 2-factors solution in an oblique EFA.

	Factor 1	Factor 2
Paper folding	0.**61**	0.15
Mental rotations	0.**59**	−0.00
Mental cutting	0.**36**	**0.43**
Schnitte	−0.00	**0.83**
figure selection	0.**49**	0.23
Cubes	**0.70**	−0.08
Factors correlation	**0.48**	

Values in bold indicate significance at $p < 0.05$.

Next, we conducted a confirmatory factor analysis (CFA) in which a single common factor was specified, as initially hypothesized. As expected given the result of the EFA, fit of this model indicated considerable misspecification (though it could be accepted): χ^2/df = 37.39/9 ($p < 0.001$); RMSEA =0.10 (90% CI = 0.07–0.13); CFI = 0.93; SRMR = 0.04. According to modification indices, the residual variances of MCT and Schnitte were highly correlated. When this correlation was freely estimated model fit significantly improved (χ^2/df = 15.85/8 ($p = 0.04$); RMSEA = 0.06 (90% CI = 0.01–0.10); CFI = 0.98; SRMR = 0.03). Parameter estimates for this model are presented in Figure S1.

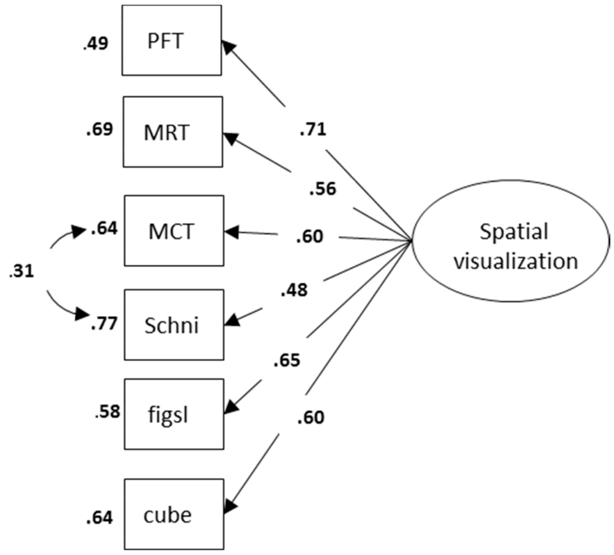

Figure. S1. CFA on SV tests. PFT=Paper Folding Test; MRT=Mental Rotations Test; MCT=Mental Cutting Test; Schni=Schnitte; Figsl=Figure selection. All paths are significant at $p < 0.001$.

The significant correlation between the residuals of Schnitte and MCT could result for several reasons. First, both tests require the visualization of cross-sections, and this type of SV might substantially differ from the types of SV involved in the other tests (e.g., mental rotations). Second, both tests had a rather loose time limit comparing to the other tests, which may have, additionally increased their covariation. The loose time limit may have increased the likelihood of using non-spatial solving strategies. Given these results, it seemed justified to retain a measurement model in which the unique variance of Schnitte and MCT was estimated and differentiated from the factor common to all of the tests. Note that both Schnitte and MCT still had high loadings on the common factor.

A Correlated Factors Model

We next conducted a CFA with the model shown in Figure 1 in the main paper. Fit indices for this model were: $\chi^2/df = 95.42/50$, $p \leq 0.001$; RMSEA (90% CI) = 0.05 (0.04–0.07); CFI = 0.94; SRMR = 0.05. Although we accepted this model, misspecification was still present. According to modification indices, "Schnitte" loaded significantly on the verbal factor, and freeing this path would have further improved model fit. Indeed, the test 'Schnitte' was likely verbally (and not only spatially) demanding, as it included complex verbal instructions and problems which were presented only verbally. However, although allowing this cross-loading could be justified, we chose to retain for further analyses the less accurate but simpler model, which was still of acceptable fit.

III. Multiple Group Invariance

Although mean differences on the observed variables was trivial, we tested whether measurement invariance existed across the groups so that predictions could be validly compared. Measurement invariance was to confirm that the ability factors as modelled above measured the same constructs in each group. We conducted multiple group analysis for the correlated factors model described above following steps described in Kline (2016) and Wang & Wang (2012). We first tested for configural invariance, which indicated whether the number of factors and pattern of factor loadings was similar across groups, without imposing equality constrains on any of the parameters. Next, we tested for scalar invariance, which indicated whether factor loadings and intercepts could be assumed similar in both groups. The results are summarized in Table S4. We also report the results for the baseline model as tested separately in each group (first two rows in Table S4). Because we used the MLR estimator, the values for the chi-square difference test are adjusted based on the Satorra-Bentler correction factor (Satorra & Bentler, 2001). As shown, fit of the configural model was satisfactory, and the scalar model did not significantly differ from the configural model. Thus, we could assume measurement invariance was achieved across groups.

Table S4. Multiple group analysis for measurement invariance.

	$\chi2$	df (p)	χ^2 Diff	df Diff (p)	RMSEA (90% CI)	CFI	SRMR
**3 Correlated Factors**							
ME only	77.89	50 (0.01)	-	-	0.06 (0.03–0.09)	0.926	0.059
MP only	73.60	50 (0.02)	-	-	0.05 (0.02–0.08)	0.945	0.059
1. Configural	151.43	100 (0.00)	-	-	0.06 (0.04–0.08)	0.936	0.059
2. Scalar	175.70	118 (0.00)	24.57	18 (0.14)	0.06 (0.04–0.07)	0.928	0.070

ME=mechanical engineering; MP=math-physics; Diff=difference; df=degrees of freedom; RMSEA=root mean square error of approximation; ndex; SRMR= Standardized Root Mean Square Residual.

IV. Analyses in Which Missing Values on Grades Were Excluded

Tables S5–S6 and Figures S2–S3 are identical to Tables 5–6 and Figures 2–3 in the main paper, except that here the results are for data in which missing values on grades were excluded from the analysis.

Table S5. Correlations between ability measures and grades among engineering students (N = 133).

		Analysis	L.Algebra	Mechanics	M.Elements	T.D.CAD
Spatial visualization	Paper Folding	0.05	0.07	0.10	0.21*	0.30***
	Mental Rotation	−0.07	−0.08	−0.04	0.09	0.24*
	Mental Cutting	0.14	0.13	0.06	0.22*	0.27***
	Schnitte	0.11	0.07	0.06	0.13	0.25***
	figure selection	0.03	0.04	0.07	0.18	0.22*
	cubes	−0.01	0.01	0.02	0.05	0.21*
Verbal reasoning	S.completion	0.04	−0.02	0.01	0.25**	0.01
	Analogies	0.12	0.13	0.17	0.30***	0.24**
	Similarities	−0.04	−0.11	−0.08	0.08	0.07
Numerical reasoning	Calculations	0.39***	0.32***	0.37***	0.25**	0.25*
	Num. series	0.25**	0.27**	0.29**	0.25**	0.06
	Num. signs	0.18*	0.22**	0.20*	0.15	0.10

*p < 0.05, **p < 0.01, ***p < 0.001.

Table S6. Correlations between ability measures and grades among math-physics students (N = 140).

		Analysis	L.Algebra	Physics I	Physics II
Spatial visualization	Paper Folding	0.14	0.23*	0.16	0.15
	Mental Rotation	0.05	0.15	0.12	0.06
	Mental Cutting	0.24**	0.34***	0.35***	0.35***
	Schnitte	0.12	0.28**	0.27**	0.31***
	figure selection	0.09	0.19*	0.14	0.12
	cubes	−0.02	0.06	−0.02	−0.03
Verbal reasoning	S.completion	0.19*	0.26*	0.40***	0.44***
	Analogies	0.21**	0.26**	0.29**	0.30***
	Similarities	0.13	0.29***	0.23*	0.21*
Numerical reasoning	Calculations	0.27**	0.35***	0.35***	0.28**
	Num. series	0.07	0.08	0.03	0.01
	Num. signs	0.17*	0.20*	0.24**	0.18*

*p < 0.05, **p < 0.01, ***p < 0.001.

Table S7. Fit statistics for measurement models in each group.

	χ^2	df (p)	RMSEA (90%CI)	CFI	SRMR
M. Engineering	119.23	95(0.05)	0.04 (0.01–0.07)	0.97	0.07
Math-physics	131.86	86 (0.001)	0.06 (0.04–0.08)	0.95	0.07

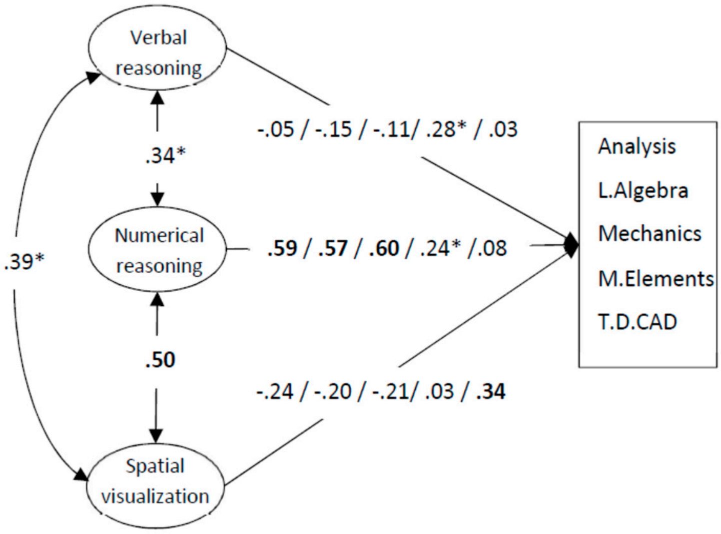

Figure S2. A SEM for predicting grades among engineering students (N = 133). On each path from abilities to grades estimate from left to right refer to grades from top to bottom. Bolded values indicate $p < 0.001$; $*p < 0.05$.

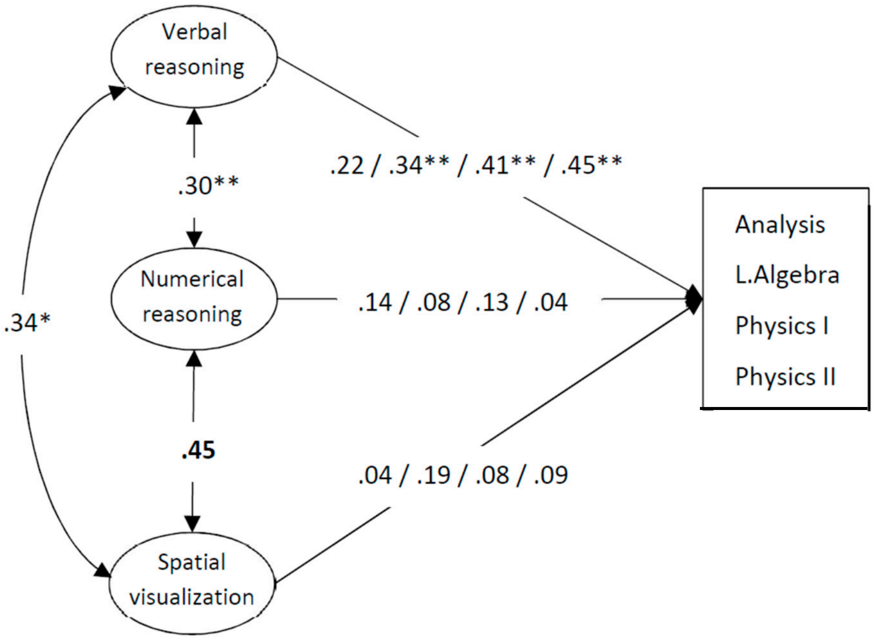

Figure S3. A SEM for predicting grades among math-physics students (N = 140). On each path from abilities to grades estimate from left to right refer to grades from top to bottom. Bolded values indicate $p < 0.001$; $*p < 0.05$.

© 2018 by the authors. Submitted for possible open access publication under the terms and conditions of the Creative Commons Attribution (CC BY) license (http://creativecommons.org/licenses/by/4.0/).
